# Informing existing technology acceptance models: a qualitative study with older persons and caregivers

**DOI:** 10.1007/s10433-024-00801-5

**Published:** 2024-03-29

**Authors:** Nadine Andrea Felber, Wendy Lipworth, Yi Jiao (Angelina) Tian, Delphine Roulet Schwab, Tenzin Wangmo

**Affiliations:** 1 Institute of Biomedical Ethics, Faculty of Medicine, University of Basel, Bernoullistrasse 28, 4056 Basel, Switzerland; 2 Department of Philosophy, Macquarie University, 25B Wally’s Walk, Sydney, NSW 2109 Australia; 3 La Source, School of Nursing Sciences, HES-SO University of Applied Sciences and Arts of Western Switzerland, Lausanne, Switzerland

**Keywords:** Technology, Caregiving, Aged care, Wearables, Sensors, Robots

## Abstract

New technologies can help older persons age in place and support their caregivers. However, they need to be accepted by the end-users to do so. Technology acceptance models, such as TAM and UTAUT and their extensions, use factors like performance expectancy and effort expectancy to explain acceptance. Furthermore, they are based on quantitative methods. Our qualitative study investigates factors fostering and hindering acceptance among older persons and their caregivers for a variety of assistive technologies, including wearables, ambient sensors at home with and without cameras and social companion robots. The goal of this paper is twofold: On the one hand, it investigates the factors of technology acceptance models in a qualitative setting. On the other hand, it informs these models with aspects currently overlooked by them. The results reveal that performance expectancy and effort expectancy are relevant for acceptance. We also find that reliability, anxiety around technology and different social aspects have an influence on acceptance of assistive technology in aged care for all end-user groups. Our findings can be used to update current technology acceptance models and provide in-depth knowledge about the currently used factors.

## Introduction

As the world population is growing older, the number of older persons in need of care is ever increasing, expecting to cross the threshold of 16% of the world population by 2050 (United Nations Department of Economic and Social Affairs 2022). At the same time, professionals who are willing to do caregiving work are becoming increasingly scarce (Santos and Miguel 2020; Seidlein et al. 2020) and informal caregivers become overburdened and distressed (Syse et al. 2022). To remedy the growing imbalance between older persons in need of care and available care, research is turning toward new technologies. These include, for example, fall detection sensors (Bet et al. 2019; Momin et al. 2022), cameras in the home (Pool et al. 2022), GPS tracking for people with dementia (Bayat and Mihailidis 2021), to cognitive assistants (Holthe et al. 2022) and artificial social companions to relieve loneliness (Gasteiger et al. 2021; Koh et al. 2021).

The actual use of new technologies necessitates investigations into factors that foster and hinder acceptance of such technologies in caregiving. Acceptance in this context matters both because developing technologies that are then rejected by end-users can be wasteful, and “forcing” interventions that people do not want is morally problematic. As engineers, scientists and politicians make choices regarding which technologies are developed and for whom and what purpose, it is crucial to ensure that these choices align with what society wants and what is within ethically acceptable boundaries (Legault et al. 2018).

Several models and theories have emerged over time to investigate and understand acceptance of technology (AlQudah et al. 2021). On established theory is the technology acceptance model (TAM), (Davis 1989). It proposes two main variables to predict acceptance: perceived usefulness and perceived ease of use (Davis 1989). Another widely used model is the unified theory of acceptance and use of technology (UTAUT) (Venkatesh et al. 2003). UTAUT consists of a combination and refinement of earlier models and has already produced a vast body of research for healthcare technologies (AlQudah et al. 2021). UTAUT uses four dimensions to predict acceptance of technology: performance expectancy, effort expectancy, social influence and facilitating conditions. Performance expectancy refers to the degree to which the user believes that using the technology can help attain goals of productivity, while effort expectancy expresses the ease (or difficulty) associated with using the technology (Venkatesh et al. 2003, p. 450). Social influence is defined as how much the end-user perceives that important others want him/her to use the technology (Venkatesh et al. 2003, p. 451). Facilitating conditions capture the extent to which they believe that the necessary infrastructure exists to support use of the new technology (Venkatesh et al. 2003, p. 453). Both UTAUT and TAM have undergone refinement over the years, and extensions of the models, such as UTAUT 2 or TAM 3, have seen the addition of new variables, such as anxiety and cost, among others (Patil et al. 2020; Rondan-Cataluña et al. 2015; Tamilmani et al. 2021; Venkatesh et al. 2012).

Researchers using these models have made important contributions to the investigation of the facilitators and barriers to uptake of technologies that can also be used in aged care. Examples of investigated technologies include mHealth services (Alam et al. 2020; Hoque and Sorwar 2017; Palas et al. 2022; Rajak and Shaw 2021; van der Waal et al. 2022), wearables (Wang et al. 2020), exergames (Xu et al. 2023) and smart home healthcare services (Kang et al. 2022). Studied demographics are older persons (Alexandrakis et al. 2020; Palas et al. 2022; Xu et al. 2023; Zeng et al. 2023), healthcare professionals (Ketikidis et al. 2012) and informal caregivers for people with dementia (Wójcik et al. 2021), among others. Many of these studies have confirmed the validity of the two models, yet also discussed factors outside of these models that may influence acceptance (Paccoud et al. 2021). To investigate the relations between the different variables of the theories, researchers mostly use surveys and carry out statistical analysis based on such quantitative data. However, some researchers have used these theories to inform their qualitative work (Drehlich et al. 2020; Ehn et al. 2019; Hanif and Lallie 2021; Vandemeulebroucke et al. 2021). Both TAM and UTAUT have, however, been criticized for their arbitrariness (Bagozzi 2007), lack of certain dimensions (Dwivedi et al. 2019) and complexity (Tamilmani et al. 2021). Regarding acceptance of technology in general, qualitative research has uncovered relevant issues that have not yet been discussed in the more quantitative approaches, such as deception, the fear of replacement of human care (Wangmo et al. 2019) or loneliness (Zsiga et al. 2013), among others. Such findings demonstrate that existing models can benefit from qualitative research.

The goal of this qualitative paper is therefore to inform these theories of technology acceptance. More specifically, we aim to (1) validate these theories beyond their usual quantitative validation, (2) provide context and depth to the dimensions of TAM and UTAUT and (3) allow the emergence of additional factors fostering and hindering acceptance of technologies to potential add new, overlooked dimensions important for technology acceptance. To achieve these goals, we are mapping the acceptance of new technologies in aged care among the three stakeholder groups (older persons, professional caregivers and informal caregivers), as well as for a variety of technologies.

## Research methodology

Exploring the conditions that facilitate and hinder the adoption of technologies in aged care by older persons and their caregivers in Switzerland was one of the main goals of the RESOURCE project. This mixed-method project involves systematic reviews of literature (Felber et al. 2023; Tian et al. 2024), qualitative interviews on which this paper is based and a quantitative national survey.

### Interview guide

The purpose of this qualitative part of the project was to explore the opinions of stakeholders living in Switzerland regarding both existing and potential technologies that could be used to care for older persons. These stakeholders were older persons, professional caregivers and informal caregivers, such as family members or friends. To capture as much data as possible while still offering a degree of consistency, the interviews were fashioned in an explorative, semi-structured manner with open-ended questions (Magaldi and Berler 2020). The interview guide was drafted based on the current literature and after consulting experts in assistive technologies for older persons. It was divided into three sections: section one explored the current caregiving-situation and its challenges; section two discussed specific technologies; section three asked general questions regarding conditions and policies facilitating or hindering the adoption of technology in caregiving, for example, “How could the acceptance of smart home technologies be improved in the future?”.

Section two was further divided into two categories: monitoring technologies and devices that were meant for entertainment and companionship. The former category included emergency wrist buttons, wearables and sensors at home (with and without video). The latter category included virtual reality (VR), the robot Pepper (a humanoid robot that is able to interact with users in a variety of ways, such as speech, demonstrating exercises and games, due to a tablet on its chest) (Tanioka et al. 2019) and the robot Paro (a social robot with the appearance of a baby seal that was developed for people with dementia (Shibata 2012)). The technologies were demonstrated through pictures and videos (in German). Participants were asked a variety of questions for each technology, for example, “What is your opinion on wearable sensors that monitor your health-related data, as well as your location (GPS), e.g. heart rate, blood sugar, sleep phases, physical activities?” or “What benefits or problems do you foresee when using wearable sensors?” They were also asked to compare the technologies among each other, with questions such as “which one of these technologies would you most likely use and why?”.

The original English interview guide was translated to German and French by the first author and then underwent back translation by two independent researchers to ensure consistency and coherence between languages. The original interview guide was then slightly adapted for each stakeholder group.

### Ethics

The research project was approved by the Ethics Commission of Northwest and Central Switzerland (EKNZ) under ID: AO_2020-00027. A document containing all relevant information regarding the purpose of the study, the content of the interviews, the measures taken to ensure data privacy and a contact person (first author) in case of doubts were distributed before the interview to each participant. Participants then had the opportunity to discuss the document with the interviewer before the interview and thereafter provided written consent. Participants were also informed that they could withdraw at any point during the interview or after the interview prior to anonymization of data. No participant chose to withdraw participation.

### Participant recruitment

Participants were recruited through a variety of techniques, including contacting relevant institutions, such as nursing homes, home care organizations and additional services (such as Meals on Wheels), distributing flyers in points of interest, such as organizations centered around age or caregiving, online advertising, for example, in online journals centered around aging, as well as social media and word of mouth. Purposive and snowball sampling were therefore combined. Conditions for inclusion were the following: (1) being 65 years or older OR being a caregiver to a person 65 years or older and (2) living in Switzerland at the time of conducting the interview.

### Data collection

Two native female German-speaker interviewers, trained in qualitative methods and interviewing skills by the last author, carried out data collection. The interviews were recorded using a small portable recorder. The first author is one of the interviewers. She was completing her PhD in biomedical ethics at the time. The other interviewer (acknowledged in the paper) was pursuing her master’s degree in medicine. The first interviewer collected 85% of the interview data, while the second collected 15%. The interviews were on average 96 min long (range: 46–189 min). The interviews were usually conducted in one session, except for cases where the participant was either pressed on time (formal caregivers) or unable to continue the interview without a break (older persons). Most of the 60 interviews were conducted individually except in seven cases: three interviews had two older persons present (married couples), one interview had two informal caregivers present (married couple), one interview had two formal caregivers present, one interview had an older person and her formal caregiver present and one had an older person and her informal caregiver present (mother and son). The participants were able to choose the location of the interview and mostly chose their home (in case of older adults and informal caregivers) or their workplace (in case of formal caregivers). Only one interview was carried out over zoom at the request of the participant.

### Study participants

A total of 60 interviews took place with 67 participants. Table 1 offers an overview of the demographics of the study participants.

**Table 1:** Demographics of participants

Older persons (OP)*	Professional caregivers (PC)	Informal caregivers (IC)
Living at home: 13	Working for home care services: 9	General caregiving: 8
Living in nursing homes: 10	Working in nursing homes: 9	Caregiving for someone with dementia: 7
Living in assisted living facilities: 4	Other: 5	Care recipient in nursing home: 2
Female participants: 15 (μ age = 90.2)	Female participants: 19 (μ age = 45.6	Female participants: 13 (μ age = 56.6)
Male participants: 12 (μ age = 84)	Male participants: 4 (μ age = 43.3)	Male participants: 4 (μ age = 58.8)

*In the result’s section, we use the following abbreviations to identify the study participants: OPH, older person living at home; OPN, older person living in nursing home; PCH, professional caregiver working in home care; PCN, professional caregiver working in nursing home; PCC, professional caregiver working in assisted living facility or smart home residence; ICR, informal caregiver for older person without dementia; and ICD, informal caregiver for older person with dementia

### Data analysis

The interviewers transcribed all interviews verbatim into German. The finished transcripts were then analyzed with the support of the co-authors (third and last authors) using thematic analysis (Braun and Clarke 2006, 2019; Guest et al. 2012). The analysis was first conducted in the group using the software MaxQDA. These group sessions served to familiarize the whole team with the data, as well as to ensure consistency in naming codes and agreeing on what each code would encompass. At least 3 transcripts for each stakeholder group were analyzed fully in this group setting. Thereafter, first author analyzed the remaining transcripts for the professional and informal caregivers, while the third author analyzed the interviews of older persons. The initial coding and theme development were conducted in such a way that they left room for reflexive thematic analysis (Braun and Clarke 2019). This method encourages reflection to reach more depth and interconnectedness in the development of themes. Themes developed after the initial sessions were brought back to the group for further sorting and refinement, until all authors agreed on the relevance and interpretation of themes and codes.

Given the qualitative nature of this article, we refrained from presenting the results in a quantitative manner. However, if a certain feature was very prevalent, for example, mentioned by all stakeholder groups, we identify the feature as such. Translated quotes from German into English are provided for illustration purposes.

## Results

In this section, we present our study findings related to features that study participants revealed as fostering and hindering acceptance of new technologies. These features are mapped onto the facets of UTAUT and TAM (as well as some facets appearing in the refined models). As the goal of our project was to discover all potential conditions fostering and hindering acceptance of technology, we conducted our analysis inductively at first, then at a later stage compared them to the currently used acceptance models. We ended up using the terminology of UTAUT for expected gain (performance expectancy) and expected effort (effort expectancy). While reliability is one of the most prevalent feature in our analysis, it is not part of the current acceptance models. We thus chose to present it as an expression of effort expectancy as unreliable technologies increase the effort needed to use them (Figalová et al. 2022). Anxiety is a variable of TAM3 (Rondan-Cataluña et al. 2015; Venkatesh et al. 2012) and is used in our analysis to capture fears related to the use of technology. Furthermore, we modified the original TAM variables of “social influence” and “facilitating conditions.” Firstly, we use the term “social aspects” to capture other social factors that go beyond the perception that important others want the person to use the technology. Secondly, we employ the term “hindering conditions” to capture non-social factors that hinder the uptake of technology. Our results are divided into four sections, first discussing features that were common to all technologies (see Tables 2 and 3 presenting common barriers and facilitators for all technologies). Thereafter, we highlight features that were unique to each of the three types of technology studied (wearables, sensors and robots).

**Table 2:** Quotes for theme “Barriers common for all technologies”

Quote 1 (Performance Expectancy)	*OPH11: And assistive technology can be used, of course, so in principle, if you…. There are beds that make it easier to get in, for example. So there are certain medical aids that are certainly useful, but I'm always happy if you can actually control everything yourself and do it yourself. Everything else is a…is an attempt to make it somewhat easier, but it certainly doesn't satisfy because you realize that you are limited*
Quote 2 (Performance Expectancy)	*Interviewer: If, let's say your daughter, came up to you and said, “We're giving you cameras now,” how would you respond? Or install for you.* *OPH4: Don’t bother.* *Interviewer: Don’t bother?* *OPH4: Yes, don't bother, I'm in the retirement home before I get cameras I'm feeling. Before at home—what should I do with this stuff? Doesn't do me any good. It says nothing, it has nothing and it cooks nothing. Nothing.*
Quote 3 (Effort Expectancy)	*OPH7: [technology needs to be] Simple, exactly. Not too complex. Not every possibility either [is needed] … So like this cell phone. When there are a lot of options, it may confuse someone. So as easy and simple as possible in terms of operation.*
Quote 4 (Effort Expectancy)	*ICD1: Yes, but someone has to be there as well, you can't just put it [Pepper] down and walk away. I can't imagine that, someone still has to be there to explain it.*
Quote 5 (Reliability)	*OPN3: How does it [wearable] notice [that I have fallen]? If I fall on it, but if the watch doesn't hit the floor, I somehow fall on my back or otherwise and am hurt, but the watch…I don't quite believe in this watch now. If I can press it [alarm button] myself, then I know now that I am making an alarm. Yes, if that [wearable] would react for sure, but I don't quite believe that what is built into it would be so good.*
Quote 6 (Reliability)	*ICR10: And I could imagine that, he [father] would probably enjoy one of those and then that somehow still entertains him. But just, it would have to function well (Pepper). If it doesn't function, there's trouble. Any lawn robot that doesn't run at its time, where it's actually programmed to run, then that can make him incredibly angry and also keep him busy and then he cannot let [the frustration] go. So you usually have to go up there on the same day and see why this lawn robot is not working as programmed. I have the feeling that you make people even more angry when it doesn't work.*
Quote 7 (Anxiety)	*ICR5: So it [technology] can be too much for them [older persons]. Especially my grandmother, she is always afraid of breaking something on her tablet or cell phone, of deleting something or… to simply destroy something irrevocably. And so she can also be very needy in that regard, sometimes.*
Quote 8 (Anxiety)	*OPN7: No, I don't see that [any advantages of robots] … Yeah, maybe…in mechanics somewhere I would see it, maybe in the factory or something, if you can do that. But not replacing it with people. I can't say yes to that.*
Quote 9 (Social Aspects)	*ICR5: Perhaps you have the idea that you can put an elderly person, equipped with sensors, cameras, robots and a stuffed animal, in a lonely apartment and say, yes, people are looking at him. That's a very exaggerated way of putting it. Because I'm a bit convinced that personal relationships are what make life worth living in the end, and what kind of life is it if you just… vegetate with technologies? You're fine because physically you're taken care of by your little robot, but mentally you're maybe a bit numbed by this robot kitten that you have and your little (robot)seal, but in the end nobody is interested in you anymore.*
Quote 10 (Social aspects)	*PCH3: Yes it would then probably be the case that you would have fewer assignments, less contact, that would then mean you would see the people less and would not capture everything else that is around.*
Quote 11 (Hindering conditions)	*ICR8: if you want to increase acceptance, then the price always comes into it and who pays that, that's also clear. Here in Switzerland, we have the philosophy that health costs us nothing. Period. (…). It's state-given and insurance-given. And that is not good*
Quote 12 (Hindering conditions)	*PCH2: Yeah we hear that so much. We can't pay that. Just already with the key lockers, which actually give a security. It always comes, it's always a question of cost.* *Interviewer: Oh, interesting.* *PCH2: How much does that cost? Or we can't pay that… So there's really a lot coming. So in every area. So even in people with dementia, where people ask me, who pays for that, do we have to pay for that ourselves? So that comes a lot. I think that's a big concern that still comes before all the benefits.*

**Table 3:** Quotes for theme “Facilitators common for all technologies”

Quote 1 (Performance Expectancy)	*OPN9: I think it [cameras] would take some of the pressure off the staff. And there is always a shortage of staff, not here in particular, I also hear it on the news on TV (…), That might not be a bad idea. That would likely save the staff some back and forth if they could track that.*
Quote 2 (Performance Expectancy)	*PCH6: Yes, that [the smartwatch] would be totally good, totally positive, in principle. But you have to look at it a little bit differentiated, so the nudging technology that reminds me, I have to take my pill in the morning, just this feature, I would want to adopt that right away, because that's exactly what we do with our appointments [home care].*
Quote 3 (Social Aspects)	*OPH11: Yes, well, I'm (unintelligible) should have that [wearable] everyone who lives alone and no second person is in the household, because you are practically then yes, yes, you are alone, you cannot help yourself. And if anything would be that that doesn't even have to be a health problem, but if you fall, for example, and you can't get up…that's a very valuable resource.*
Quote 4 (Social Aspects)	*PCH4: That's really difficult to say. I think it also depends on whether I would live alone or whether there is still a partner, where you are maybe two in this apartment, but that is anyway probably decisive in all these points, whether I would be alone or not.*
Quote 5 (Facilitating conditions)	*OPH1: I think if you wanted to introduce this, you don’t have to talk about it, you have to put it in front of people and try it and then everyone says “oh that’s handy.” I never wanted a smartphone until someone said, I have one, it costs 50 bucks, now take it and just try it out. And of course, now I am hooked and very happy and don’t do anything without the thing.*
Quote 6 (Facilitating conditions)	*ICD3: I think we simply have to educate people much more, so that they also see that a robot can be a help in a nursing home, for example. Yes, so I think the education.*

### Barriers common for all technologies

#### Performance expectancy

Although there was no consensus across all data, utility and necessity (or lack thereof) were themes that emerged across all stakeholder groups and in relation to all technologies. For the most common explanation given for the uselessness of technology was the fact that the participants can also live their life like their parents and grandparents who were fine without such technologies. Even where it was acknowledged that technologies could make life easier, this was not necessarily seen as a good thing. One participant noted that while technology might have utility, he would rather have the ability to function without it. For some technologies, participants weighed the benefits against the costs and often concluded that the negative impacts outweighed the positive consequences.

#### Effort expectancy

Participants from all groups expressed the view that technologies are not designed for older persons—particularly because of their complexity. Participants articulated what it meant for a technology to be sufficiently simple. For one older participant, “simple” meant having options that are not too many to be confusing and ease of using the technology. Underscoring the complexities of existing technologies, an informal caregiver discussed how difficult it could be even for their generation to keep up to date with technological nuances. Moreover, caregivers from both groups feared that their older care recipient would be overwhelmed by a new device, not being able to handle its complexity, and therefore rely on their caregivers for help whenever the technology would cause issues, thus increasing their caregiving burden. Robots that are meant to be used autonomously by the older persons especially received skepticism regarding increased caregiving burden, as caregivers were not convinced that older persons would be comfortable using the robot without any assistance.

Participants from all groups had concerns regarding the reliability of the discussed technologies, relaying concerns with each of them. There were, for example, worries expressed about false alarms and that the call for support in emergency may not function when needed. With regard to robots, caregivers expressed that a malfunctioning robot would upset their care recipient and that caregivers would be asked to fix issues immediately. In the case of monitoring technologies, all groups reported problems with charging the device, which may result in older persons forgetting to wear the device consistently.

#### Anxiety

Both formal and informal caregivers discussed how their care recipients could become anxious when using technology and that their confidence often remains low regarding the use of devices. Older persons did not explicitly mention that new devices would make them anxious or that they are not confident in using them. However, they often revealed general reluctance toward any technology that was presented to them as a first reaction. This was especially prominent for robots, where older persons disliked interacting with artificial beings and sometimes even imagined dystopian scenarios where robots would either replace all human caregiving or go rogue. Caregivers from both groups too imagined that older persons could be distressed or anxious when using robots. Furthermore, older persons were worried about technology’s reliance on electricity and the additional waste produced. At least one participant from each stakeholder group mentioned worries about radiation.

#### Social aspects

All participant groups mentioned that old persons may just be too old to introduce technology into their lives, suggesting that future generations will adopt caregiving technologies more easily because of previous exposure. Furthermore, the participants mentioned how they prefer human caregiving rather than caregiving facilitated by technology. This seemed especially true in regard to robots, as a few participants could envision robots as social companions. Formal caregivers worried that monitoring technologies would diminish the social, interactive aspect of caregiving and that the care recipient would receive less attention, as only data would be used to assess the patient.

#### Hindering conditions (digital literacy and cost)

Older persons especially complained that they were not informed about technological or digital innovations. Similarly, the caregivers were also surprised at their lack of awareness of technologies that could be used in a caregiving context. Another aspect mentioned by stakeholders that negatively influenced acceptance were most often cost of these technologies.

### Facilitators common for all technologies

#### Performance expectancy

Participants from all groups felt that some technologies would alleviate caregiving burden. The possibility of technology relieving caregivers (both formal and informal) increased acceptance for all participant groups. Participants easily imagined scenarios where technology could offer a benefit, and many mentioned the shortage of human caregivers or their lack of expertise as a reason why technology should be used in caregiving.

#### Social aspects

A social condition that seemed to increase acceptance was living alone. Participants from each stakeholder group mentioned that they would find the use of technology more acceptable if they or the care recipient were living alone, as they imagined themselves being more vulnerable to accidents and therefore needing a device to alert someone in case of a fall.

#### Facilitating conditions

Mirroring the hindering condition of not knowing about technological support and cost, participants from all groups wished for more education around technology as well as financial support. Trying out the technology or seeing the benefits first hand was often mentioned as important facilitators by all stakeholder groups.

### Barriers to acceptance of wearables

#### Performance expectancy

We asked participants about wearables that would dispatch automatic alarms in case of a fall and that would monitor their health data (such as sleep, heart rate and steps and potentially blood pressure and other more sophisticated readings in the future) and monitor their location (via GPS). While older persons expressed general lack of interest in wearables, many caregivers specified that health data are not useful in and of itself, as more information is needed to assess how the care recipient is doing (Tables 4 and 5). Specifically, the older person should rather be asked herself how she feels, to get a proper assessment of their status. Informal caregivers felt that the health data would overwhelm them, and that they would not appreciate the added responsibility of checking the health data (Table 5).

**Table 4:** Quotes for theme “Barriers to acceptance of wearables”

Quote 1 Effort expectancy	*ICR10: Yeah I don’t think they [older parents] could read that and they wouldn’t understand that [wearable]. So for myself I do that, I sleep like that with the watch and I count my steps and stuff, so for me personally I think it’s great. I still understand it though. (…) But I don’t know if I would want that from my parents. And I think I could do too little with it. I would almost have to send it to the doctor, you know, if I were to see you, Mom, you would have to go to the doctor now, because somehow…. and I don’t want that. No, no. But if they had that and it went straight to the doctor, then I would think it's good too…* *I don’t want to have to take that responsibility, you know?*
Quote 2 Effort expectancy	*PCC1: Every wearable now that comes on the market, they are getting smaller and the buttons are really so flat and sensors, I find that is so tactile, that is not good at all for older people. They need a device that looks like a watch but where you can still press the button*
Quote 3 Effort expectancy	*OPN10: You can’t press it, at least not well. I don’t know how the others do it. Two or three have already said, “I can’t get do it. I can’t get do it."*

**Table 5:** Barriers and facilitators to acceptance of wearables

Group	Older participants	Professional Caregivers	Informal Caregivers
Barriers	Performance expectancy *Lack of interest* *Satisfied with own somatic assessment*	Performance expectancy *Data does not give a comprehensive understanding of the patient*	Performance expectancy *Lack of interest in health data of older persons*
	Effort expectancy Reliability (e.g. false alarms) *Certain cognitive capacities needed* *Design* *Dislikes aesthetic*	Effort expectancy Reliability (false alarms, inaccurate measurements, charging) *Certain cognitive capacities needed* *Design* *Complexity* *OP will dislike aesthetic*	Effort expectancy Reliability (false alarms, charging) *Certain cognitive capacities needed * *Design* *Complexity* *OP will not like aesthetic*
	Anxiety Data causing nervousness/pressure/hypochondriac	Anxiety Data causing nervousness/pressure/hypochondriac	Anxiety Data causing nervousness/pressure/hypochondriac
		Social aspects Focus on data detrimental to relationship	
Facilitators	Performance expectancy Reassurance (receive help when needed, health data monitoring) *Nudging (reminders for appointments and behaviors appreciated)*	Performance expectancy Reassurance (receive help when needed, health data monitoring, locating) Increased independence of older persons Optimization of caregiving (detection of anomalies, more knowledge about older person’s condition) *Nudging (reminders for appointments and behaviors appreciated)*	Performance expectancy Reassurance (receive help when needed, locating) Optimization of caregiving (detection of anomalies, more knowledge about older person’s condition) *Nudging (reminders for appointments and behaviors appreciated)*
	Effort expectancy *Always available (if worn)*	Effort expectancy *Always available (if worn)* *Less invasive*	Effort expectancy *Always available (if worn)*
	Social aspects *Nonstigmatizing aesthetics*	Social aspects *Nonstigmatizing aesthetics*	Social aspects *Nonstigmatizing aesthetic*s

Italicized information is unique to this technology and is discussed in the content below. The other contents are presented in the general section above

#### Effort expectancy

Design came out as a specific barrier to acceptance for wearables regarding the effort to use them. In both caregiver groups, participants reported that older persons would not be able to handle complex devices with many functions and that font size, contrast and small letters may cause issues for people who have deteriorating abilities to see or hear (Tables 4 and 5). Additionally, vibrations may not be helpful in light of reduced sensitivity due to health conditions. Some older persons who had experienced more modern devices than familiar emergency buttons claimed that they are hard to use. Furthermore, all groups mentioned that the current aesthetic of wearables does not appeal to older persons, which they confirmed.

### Facilitators of acceptance of wearables

#### Performance expectancy

The main expectation all participant groups had from wearables was reassurance (Tables 5 and 6). Both caregiver groups spoke of the reassurance of being able to locate a care recipient with dementia, as well as receiving health data from them when they cannot communicate their ailments well. Older persons appreciated the knowledge that they could get help when needed. Also, participants from all groups saw nudging as a useful mechanism to be reminded of appointments or healthy behavior, such as medication intake, thus enabling better self-management.

**Table 6:** Quotes for theme “Facilitators of acceptance of wearables”

Quote 1 Performance expectancy	*ICD2: And last fall she fell down somewhere and had severe knee pain. And that would also be something, for example, if she doesn’t remember to tell me such things in the future, because if I know, then I can address it with the family doctor, because at the family doctor I always go along and see that everything works out there and she, yes, she doesn’t like to remember everything.*
Quote 2 Performance expectancy	*OPN9: Yeah, and that I know then and then, and that they might alert me with a sensor, “It’s two-thirty. You have to be downstairs at a quarter to three,” for example. On the whole, I do get a message when I have an appointment, but it doesn’t always work out. And that’s where sensors would be a good idea. I don’t know how it works, but a suggestion.*
Quote 3 Social aspects	*OPH7: It was just so clunky [emergency button]. You immediately see, ah yes, this is an old person who now needs such a watch. But now, I think it’s become better in terms of design.*
Quote 4 Social aspects	*ICR7: (laughs) Good question… I think it’s a question of amount [how many times]. If he…. would get too many reminders, then I don’t think he (father) would want that. So you’d have to be able to control it quite individually. He would probably have to be able to say what he wants to be reminded about.*

#### Social aspects

Some participants pointed out that wearables may appeal to older adults because wearing them would not be perceived as stigmatizing, because young and health conscious people are also using them (Tables 5 and 6). Caregivers suggested that wearables be fully customizable, regarding what values are monitored and what nudges are given, as well as when and how, so that older persons can adapt the device to their needs. Furthermore, customizability would ensure that older persons do not feel embarrassed because of unwanted nudges in public, as they could silence nudges according to their wishes.

### Barriers to acceptance of ambient sensors

#### Effort expectancy

From all groups, participants mentioned that sensors would be hard to incorporate into the home of their care recipient, especially because some lived in very big apartments or houses (Tables 7 and 8). Both caregiver groups and older persons foresaw sensors as more useable in newly built apartments. When cameras were mentioned, caregivers from both groups worried that their caregiving burden would actually increase with the technology, given that someone needs to watch the produced footage.

**Table 7:** Quotes for theme “Barriers to acceptance of ambient sensors”

Quote 1 Effort expectancy	*ICR2: Yes, I think that is very difficult to implement (sensors in the house). So their apartment has two floors. It is still the same place where we grew up. It has many rooms, so they could go into one room and there it has no sensor. So difficult to implement.*
Quote 2 Effort expectancy	*ICD5: So I imagine a video camera that films 24 h and then you want to see if something has happened. But you don’t have the time, that’s not possible doesn’t always work out. And that’s where sensors would be a good idea. I don’t know how it works, but a suggestion.*

**Table 8:** Barriers and facilitators to acceptance of ambient sensors

Group	Older persons	Professional caregivers	Informal caregivers
Barriers	Performance expectancy *Too intrusive for home* *Living in nursing home more acceptable than using cameras* *Not needed due to regular check-ins from nursing home staff*	Performance expectancy *Too intrusive for home* *Living in nursing home more acceptable than using cameras* *Not needed due to regular check-ins from nursing home staff*	Performance expectancy *Too intrusive for home* *Not needed due to regular check-ins from nursing home staff*
	Effort expectancy *Too much work to install*	Effort expectancy *Too much work to install* *Time burden of watching footage*	Effort expectancy *Too much work to install* *Time burden of watching footage*
	Anxiety Enables abuse (data theft, video theft, espionage for burglaries)	Anxiety Enables abuse (data theft, video theft, espionage for burglaries)	Anxiety Enables abuse (data theft, video theft, espionage for burglaries)
Facilitators	Perceived usefulness *Safety (cameras provide proof of abuse, burglary)*	Perceived usefulness *Safety (proof in case of disputes)*	Perceived usefulness *Safety (cameras provide proof of abuse, burglary)*
	Effort expectancy *Passive and unobtrusive*	Effort expectancy *Passive and unobtrusive*	Effort expectancy *Passive and unobtrusive*
	Social aspects *Only acceptable for people with dementia*	Social aspects *More acceptable for people with dementia*	Social aspects *More acceptable for people with dementia*

Italicized information are unique to this technology and are discussed in the content below. The other contents are presented in the general section above

### Facilitators of acceptance of ambient sensors

#### Performance expectancy

While the idea of surveillance made participants in general uncomfortable and many were worried about data abuse, in each stakeholder group at least some participants appreciated the idea of surveillance for safety purposes or proof that others are fulfilling their roles and acting ethically (Tables 8 and 9). For example, informal caregivers mentioned situations where they wished to monitor the performance of formal caregivers as they were worried about misconduct, while formal caregivers thought of situations where cameras could actually prove their innocence.

**Table 9:** Quotes for theme “Facilitators of acceptance of ambient sensors”

Quote 1 Performance expectancy	*ICR1: That's [cameras] what my friend has for her house too [cameras]. She had surgery in [neighboring town] the other day and then she had to spend the night there. And in the middle of the night there was a beep and then she sees two burglars running through her house and helping themselves. Then she called her son, but of course it was all too late anyway. But I think it's great that that's possible.*
Quote 2 Performance expectancy	*PCN7: I think it would have some small advantages, especially for someone who has late state dementia and always moves a lot of things around and has a mania of stealing …, they've already forgotten where they hid it and then you don't see it, of course, then you've been stolen from.*
Quote 3 Effort expectancy	*ICD2: And my mom, you can't see these sensors. And she wouldn't understand that that's there. So she wouldn't care at all. And for me personally, I don't really see any big disadvantages, so that's where I spontaneously… So I see it more as an advantage, just, if something happens, that then it can be reacted accordingly and can also be reacted quickly, maybe that's what it comes down to.*
Quote 4 Social aspects	*ICD3: For me, the button and the watch are the most likely, because if I imagine that someone is watching me *via* the camera, I wouldn't find that so thrilling (laughs). Unless I assume that I would then have dementia, then I would probably also be happy about such sensors, that, if I am not at home, someone is looking for me.*

#### Effort expectancy

Despite expressing that sensors at home would be difficult to install, informal caregivers appreciated the fact that, once set up, the care recipient would be monitored passively and without any effort (Tables 8 and 9). Issues that were mentioned in relation to wearables, for example, the need to remember to wear the device or to charge it, were resolved in the case of installed sensors.

#### Social aspects

Acceptance increased when study participants thought about the care recipient as being very ill or suffering from cognitive decline (Tables 8 and 9). Once the relationship between the caregiver and the care recipient changes into a more dependent stage, monitoring becomes more acceptable, possibly because of an increased worry for caregivers and thus an increased need for reassurance. Nevertheless, caregivers wanted their care recipients to live their life as normally as possible, despite their increased dependency, and therefore preferred sensors without cameras over more intrusive monitoring methods. Furthermore, older persons themselves thought that video surveillance is only acceptable for people suffering from advanced cognitive decline, and never imagined themselves as being in such a situation in the future.

### Barriers to acceptance of robots

#### Performance expectancy

For both robots presented, participants in all groups expressed the view that they were not advanced enough yet to provide any real benefit (Tables 10 and 11). For the humanoid robot, they thought it would not be able to do any useful chores, both regarding household tasks and caregiving tasks (referred in the table as instrumental tasks). For the pet-like robot, they suggested that it should at least simulate a will of its own and ask for attention, in order to provide a more interesting, real interaction that would make the older persons feel needed and useful. Interestingly, while some caregivers considered robots as being entertaining for older adults, some caregivers from both groups even rejected that use, stating that older adults should not be stimulated and entertained all the time.

**Table 10:** Quotes for theme “Barriers to acceptance of robots”

Quote 1 Performance expectancy	*OPN10: The nurse sees when I have a seed, let’s say “sand" [in my eye], she can remove it, but a robot doesn't see that. That's what I find, the little things and they are not taken into account. That's also the important thing. How can it wash my intimate parts?… But it's just these little things that are also part of it, it's all human.*
Quote 2 Performance expectancy	*ICR5: When the robot demands again, “stroke me.” So then the robot would have to meow a bit and cry for attention. And if she [grandmother] doesn't react, that he stops again, yes (laughs). Otherwise she'll throw him out of the window! (laughs)* *Interviewer: (laughs) Yes ok, but that he could behave autonomously a little bit?* *ICR5: Yes, so that it is not purely dependent on our grandma, that she only leaves the kitten on with the switch when she feels like it, because that would perhaps make the whole thing more authentic.*
Quote 3 Social aspects	*Interviewer: (Video Pepper) You're shaking your head already?* *OPH2: You get so childish when you're that old.* *Interviewer: So childish? What did you say, one becomes childish?* *OPH2: One becomes childish, yes. So when you're a kid, you might still enjoy stuff like that, but it's so unrealistic.* *Interviewer: Exactly, it's unrealistic.* *OPH2: Absolutely no, it's not like I find it entertaining. It's not entertaining or educational. Now, if it's something that can inspire you, then I'll watch that kind of show, but if it's just to pass the time, or how do you say it, to kind of entertain you or cheer you up, what…* *Interviewer: No.* *OPH2: So I prefer to look for something to read.*
Quote 4 Social aspects	*PCN5: And with Paro, I could imagine the topic of just ridiculousness…or that maybe you'd like it but you don't want to show yourself like that, that you just use something like that in your room because you're a little embarrassed.*

**Table 11:** Barriers and facilitators to acceptance of robots

Group	Older persons	Professional caregivers	Informal caregivers
Barriers	Performance expectancy *Not advanced enough (not helpful, not autonomous/real)*	Performance expectancy *Not advanced enough (not helpful, not autonomous/real)* *Older persons should not be overstimulated*	Performance expectancy *Not advanced enough yet (not helpful, not autonomous/real)* *Older Persons should not be overstimulated*
	Effort expectancy Reliability	Effort expectancy Reliability *Older Persons cannot use it alone*	Effort expectancy Reliability *Older Persons cannot use it alone*
	Anxiety *Artificial* *Robot could go rogue/disobey*	Anxiety *Artificial* *Robot could go rogue/disobey*	Anxiety *Artificial* *Robot could go rogue/disobey*
	Social aspects *Stigmatizing/infantilizing*	Social aspects *Stigmatizing/infantilizing*	Social aspects *Stigmatizing/infantilizing*
Facilitators	Performance expectancy *Entertainment* *Acceptable for instrumental tasks*	Performance expectancy *Entertainment* *Acceptable for instrumental tasks*	Performance expectancy *Entertainment* *Acceptable for instrumental tasks*
	Social aspects *Better than no or bad human care* *Acceptable for personal hygiene tasks* *Appropriate in nursing home/for groups*	Social aspects *Better than no care* *Acceptable for personal hygiene tasks* *Appropriate in nursing home/for groups*	Social aspects *Better than no care* *Acceptable for personal hygiene tasks* *Appropriate in nursing home/for groups*

Italicized information are unique to this technology and are discussed in the content below. The other contents are presented in the general section above

#### Social aspects

Participants from all groups expressed that older persons may feel judged when seen interacting with a robot (Tables 10 and 11). Robots were perceived as childish by some participants, and more appropriate for people with dementia. Older persons thought that they did not provide any form of useful stimulation or education, alluding to the fact that they still wanted to learn and expand their horizon, rather than being just entertained or occupied.

### Facilitators of acceptance of robots

#### Performance expectancy

Some participants from each group saw robots as entertaining and stimulating for older persons (Tables 11 and 12). Humanoid robots were deemed as an interesting, exciting experience, a new form of interaction. Pet-like robots could provide companionship without the burdens that come with taking care of an animal, according to caregivers from both groups. They mentioned how some older persons would still like to have pets as companions, but are unable to take care of them, thus seeing an opportunity for robots to fulfill the needs of companionship without pushing older person’s beyond their capabilities.

**Table 12:** Quotes for theme “Facilitators of acceptance of robots”

Quote 1 Performance expectancy	*OPH9: Yeah so if you ask me personally then I would say I wouldn't find that bad. I think it's a bit of a detachment, a bit of entertainment…. And then it forces you to think a little bit. A little bit, because then maybe he doesn't give an answer to what you ask him, but to what he has in mind. No, no, why not?*
Quote 2 Performance expectancy	*ICR5: And that’s all not necessary with such a little robot [feeding, *etc*.], you only have the pleasant stuff of stroking and purring and that you have the feeling that you are not so alone. I don't know whether this robot then also wakes up on its own and meows again or so. That would be great, of course.*
Quote 3 Social aspects	*OPH1: I think that it is actually always better to have a nice Pepper than an unpleasant person. So it depends on the quality of the living individual and unfortunately there are many people in the nursing home who work because of the money and you can see that. And I think that if I had to choose, I would rather have the Pepper than someone who says that the money is good, I'll do it, but after that, I don't really give a damn.*
Quote 4 Social aspects	*PCC5: And above all, let's be honest, especially in a retirement and nursing home, that's perhaps less the case here now, because they still live here independently, can go out independently…. (…)* *Interviewer: And here, in this context [independent apartments]?* *PCC5: Here in this context, if he [Pepper] were to visit selectively, then perhaps that could be done, but I mean we are here in independent living, if we plan something here now, then there is a group here that takes on the topic and is responsible for the interaction. I would see that less here now. But he is allowed to visit us (laughs).*

#### Social aspects

Participants from all groups expressed greater acceptance of robots if caregivers were unavailable or, in the case of older persons, unpleasant. Furthermore, some participants from all groups imagined that using a robot for tasks related to hygiene may actually be less shame inducing than interacting with a human caregiver. Participants from both caregiver groups saw the use of robots as being more appropriate in a nursing home setting rather than for older persons still living at home. Some older persons living at home expressed the same preference. In all participant groups, suggestions were raised about how exactly Pepper would be interacting with residents in a nursing home. For example, professional caregivers thought that robots could provide prompts to participants, reminding them of mealtimes or activities that were about to commence. Informal caregivers imagined the robot being used in group activities, discussing a certain subject prompted by Pepper and thus facilitating group interaction.

## Discussion

As mentioned in the introduction, the goal of this paper is to add qualitative research to the more quantitative models of acceptance, such as UTAUT (Venkatesh et al. 2003) and TAM (Davis 1989) in order to provide more depth and context to the main facets of technology acceptance discussed in both models, as well as to potentially reveal additional important factors that could improve these models. Thus, we categorized our data similarly to the main variables of these acceptance models. Our study revealed many barriers and facilitators that are similar to the facets of TAM and UTAUT. Nevertheless, we also found additional facets that may be worth including in future acceptance models.

In the context of the current literature, our study substantiates the acceptance models’ assumption that the easier a technology is to use and the more useful it seems to users, the likelihood of acceptance increases (Cimperman et al. 2016; Mao et al. 2015; Tubaishat 2018). Furthermore, there is evidence that the worry of caregivers that older persons would get confused by new technologies is justified and that technologies need to be simple (Chung et al. 2021; Cullen et al. 2022). Reliability is rarely mentioned as a factor in TAM or UTAUT studies (Zeng et al. 2023) and more often mentioned in studies investigating acceptance outside of the TAM and UTAUT models (Dorsten et al. 2009; Gagnon-Roy et al. 2017). Given the explicit and recurrent mention of reliability issues by our stakeholders, we suggest to include it as a predictor for effort expectancy.

Anxiety emerged as a variable in TAM 3, trying to capture the nervousness, apprehensiveness and general discomfort users experience when starting to use a technology and the variable has gained attention in research employing TAM 3 and other models (AlQudah et al. 2021; Dai et al. 2020; Khaksar et al. 2019; Meuter et al. 2003; Rajak and Shaw 2021). The caregivers in our study noticed anxiety in older persons, thus supporting the inclusion of that variable into the acceptance models. Not included within the existing models, our study uncovered that many participants expressed concerns regarding radiation, use of electricity and the production of waste (Li et al. 2019). The artificial character of robots and the fear of them disobeying also seemed to induce anxiety in study participants. Another study found that older persons prefer to limit the degree of autonomy of robots (Scopelliti et al. 2005), a preference that could be related to the fear of disobedience by robots. Furthermore, a fear that was mentioned often and by all participant groups was the replacement of human caregiving through technology. This fear has been found in other studies (Felber et al. 2023; Vandemeulebroucke et al. 2021; Wangmo et al. 2019). In order to gain more understanding of the variable labeled “anxiety” in the acceptance models, we suggest examination into new factors that we found such as radiation, fear of disobedience and replacement of human care.

We included social aspects as a variable in our analysis to capture conditions related to the living situation of older persons, their social image or interactions with others which would influence acceptance of technology. We found that living alone was deemed to foster acceptance of technology. As social influence can also be characterized as understanding the importance of adopting the technology by the targeted user (Rajak and Shaw 2021), living alone may be a factor that positively influences that understanding. Regarding the issue of feeling stigmatized by others when using technologies, especially robots, has been found by other researchers, who used also proposed to add stigmatization under social aspects influencing acceptance (He et al. 2022). We also saw that acceptance of monitoring technology increases for caregivers when the care recipient’s cognitive capabilities decline. This relationship has been found in other studies involving caregivers and people with dementia (White and Montgomery 2014; Williamson et al. 2017). As with the variable of anxiety, we therefore propose to additionally study these dimensions of social aspects so that they can also be added to the acceptance models.

Facilitating conditions such as increased education for technologies was also found by other studies using TAM and UTAUT (Liu et al. 2015; Prayoga and Abraham 2016; van der Waal et al. 2022). Turning this aspect around, the lack of awareness is an issue for the uptake of innovations in healthcare in general (Watkinson et al. 2021). Lastly, cost has been found to be an important barrier in other studies (Dai et al. 2020; Dorsten et al. 2009), and it is part of the updated UTAUT model (Venkatesh et al. 2012). However, lack of awareness has not yet found its way into the acceptance models, although some studies ask for the degree of familiarity with technology (Harris and Rogers 2023; Scopelliti et al. 2005), which is a related question. If a person is not familiar with a technology, she is also not aware of the possibilities offered by the technology.

## Future research and implementation

A next potential step could be to investigate how these barriers and facilitators can be employed to provide better caregiving with the assistance of technology. The framework of responsible innovation (RI) deals with a similar question, as one definition of RI describes it as “a transparent, interactive process by which societal actors and innovators become mutually responsive to each other with a view to the (ethical) acceptability, sustainability and societal desirability of the innovation process and its marketable products (in order to allow a proper embedding of scientific and technological advances in our society)” (Schomberg 2013, p.19). Under this definition, society defines, at least partially, which technologies are acceptable and desirable for its participants. While the detailed decisions will come down to the individuals and will differ case by case, the idea of individualization and autonomous decision making (conditions that were affirmed by study participants) need to be agreed upon on a societal level, and then respected by the designers of said technologies. What the different definitions of RI have in common is the assumption that societal participation in innovation will lead to outcomes that are not solely beneficial in an economic sense, but also promote “goodness” overall (for example, for the environment or society in general), which in turn means the involvement of societal actors to determine what this goodness exactly is (Timmermans and Blok 2021). Our study has provided a first step regarding said determination.

## Strengths and limitations

Overall, our study has shown that qualitative research investigating acceptance of technologies can add valuable information to established quantitative measures, such as the TAM and UTAUT models and their modifications. Qualitative findings are often rich and nuanced due to its qualitative nature and can provide deeper insights on the understandings of certain terms widely used in a quantitative method (Lipworth et al. 2010; Mallinson 2002). The large scope of our research ensures that our paper lays important groundwork for future quantitative research. Despite our relatively high sample size, our findings are not generalizable. Furthermore, social desirability bias may have influenced our results, as study participants may have answered questions in accordance to what they thought the authors would want them to answer (Bergen and Labonté 2020). While interviewers made an effort to avoid language that would encourage such bias, its presence cannot be excluded.

## Conclusion

This paper presents the barriers and facilitators of new technologies in caregiving as perceived by the three main stakeholders—older persons, professional caregivers and informal caregivers. Our qualitative approach found similar variables like those used by TAM and UTAUT, such as performance expectancy, effort expectancy, social influence and facilitating conditions, thus strengthening the models. It also found important nuances to these variables, such as reliability regarding effort expectancy or the fear of radiation regarding the variable of anxiety, which is a variable of TAM 3, thus suggesting how these models could be enriched. Furthermore, as our study included a variety of technologies, ranging from wearables to sensors to robots, we uncovered details regarding the individual features of the technologies that foster or hinder acceptance, such as the issues of charging regarding wearables or the perceived high effort to installing sensors at home. The results provide important insight into the acceptance of technology in aged care and can inform future research and design of technology in order to increase acceptance among the stakeholder groups.

## Data Availability

The used interview guides and transcripts are available upon request from the first author, nadine.felber@unibas.ch.
